# Dietary Inflammatory Index and Sleep Quality in Southern Italian Adults

**DOI:** 10.3390/nu11061324

**Published:** 2019-06-13

**Authors:** Justyna Godos, Raffaele Ferri, Filippo Caraci, Filomena I. I. Cosentino, Sabrina Castellano, Nitin Shivappa, James R. Hebert, Fabio Galvano, Giuseppe Grosso

**Affiliations:** 1 Department of Biomedical and Biotechnological Sciences, University of Catania, 95123 Catania, Italy; fgalvano@unict.it (F.G.); giuseppe.grosso@unict.it (G.G.); 2 Oasi Research Institute - IRCCS, 94018 Troina, Italy; rferri@oasi.en.it (R.F.); carafil@hotmail.com (F.C.); fcosentino@oasi.en.it (F.I.I.C.); 3 Department of Drug Sciences, University of Catania, 95125 Catania, Italy; 4 Department of Educational Sciences, University of Catania, 95124 Catania, Italy; sabrinacastellano@hotmail.it; 5 Cancer Prevention and Control Program, University of South Carolina, Columbia, SC 29208, USA; shivappa@email.sc.edu (N.S.); JHEBERT@mailbox.sc.edu (J.R.H.); 6 Department of Epidemiology and Biostatistics, Arnold School of Public Health, University of South Carolina, Columbia, SC 29208, USA; 7 Connecting Health Innovations LLC (CHI), Columbia, SC 29201, USA

**Keywords:** sleep quality, cognitive decline, dementia, mental health, cohort, Italy, dietary inflammatory index, diet, inflammation, dietary patterns

## Abstract

Background: Current evidence supports the central role of a subclinical, low-grade inflammation in a number of chronic illnesses and mental disorders; however, studies on sleep quality are scarce. The aim of this study was to test the association between the inflammatory potential of the diet and sleep quality in a cohort of Italian adults. Methods: A cross-sectional analysis of baseline data of the Mediterranean healthy Eating, Aging, and Lifestyle (MEAL) study was conducted on 1936 individuals recruited in the urban area of Catania during 2014–2015 through random sampling. A food frequency questionnaire and other validated instruments were used to calculate the dietary inflammatory index (DII^®^) and assess sleep quality (Pittsburg sleep quality index). Multivariable logistic regression analyses were performed to determine the association between exposure and outcome. Results: Individuals in the highest quartile of the DII were less likely to have adequate sleep quality (odds ratio (*OR*) = 0.49, 95% CI: 0.31, 0.78). Among individual domains of sleep quality, an association with the highest exposure category was found only for sleep latency (*OR* = 0.60, 95% CI: 0.39, 0.93). Conclusions: The inflammatory potential of the diet appears to be associated with sleep quality in adults. Interventions to improve diet quality might consider including a dietary component that aims to lower chronic systemic inflammation to prevent cognitive decline and improve sleep quality.

## 1. Introduction

Inflammation represents an important physiological defense mechanism that protects the human body from external insults. As long as the inflammatory response is properly regulated—in response to real insults and counteracted by negative feedback mechanisms—it remains an essential mechanism for the maintenance of body homeostasis [1]. However, current evidence supports the central role of a subclinical, low-grade systemic inflammation in a number of chronic illnesses [2]. Elevated circulating levels of markers of inflammation, including C-reactive protein (CRP), tumor necrosis factor (TNF)-alpha, and interleukin (IL)-1 and IL-6, may lead to inflammation of the central nervous system, which has a role in the progression of chronic neurodegenerative disease [3]. Consequently, there is a growing body of research investigating whether such markers prompted by inflammation might also contribute to their pathogenesis [4]. Pathological alterations in sleeping behaviors and inflammatory disease states have common origins that involve an increased number of inflammatory cytokines [5]. Metabolic and immunological consequences of sleep deprivation seem to lead to antioxidant imbalance, including perturbations in catalase and glutathione peroxidase levels, as well as indexes of glutathione recycling activities, which are decreased after sleep deprivation, while recovery sleep normalizes antioxidant content and enhances enzymatic antioxidant activities [6]. A similar relation to circulating levels of inflammatory biomarkers has also been observed with affective and cognitive disorders [7]. Several biomarkers of inflammation and antioxidants, such as CRP, γ-glutamyl transferase (GGT), carotenoids, uric acid, vitamin C, and vitamin D, have been associated with sleep quality parameters and duration [8,9].

Inflammation has been hypothesized to be a link that mediates diet and chronic diseases [10]. There is consistent evidence suggesting that "Western-like" dietary patterns, characterized by high intake of processed, refined foods, tend to be positively associated with biomarkers of inflammation, predominantly CRP, while vegetable- and fruit-based or "healthy" patterns tend to be inversely associated [11]. Evidence on the relation between diet and low-grade inflammation has been further strengthened following comprehensive analyses of intervention trials showing that a healthy dietary pattern was associated with significant reductions in CRP [12]. Among the various investigated dietary patterns, the Mediterranean diet has been associated with lower concentrations of inflammatory biomarkers (including CRP and IL-6) [13].

The Dietary Inflammatory Index (DII^®^) is a literature-derived score that has been developed to evaluate the inflammatory potential of the diet and link diet to inflammation. It takes into account six inflammatory markers (i.e., CRP, IL-1beta, IL-4, IL-6, IL-10, and TNF-alpha) [14]. The DII has proven to be of value for its association with health status in the general population [15,16,17]. Along with 18 other construct validations that use circulating inflammatory biomarkers, it has been tested for validity in a population living in the Mediterranean area [18]. Recent studies showed a potential association between adherence to a Mediterranean diet and parameters of sleep quality [19,20,21]. However, no study focusing on the relationship between the DII and sleep has been published (though one study in Korea [22] did consider sleep when assessing the relationship between the DII and cognitive decline in older adults). Thus, the aim of this study was to test the association between DII scores and sleep quality in a cohort of Italian adults.

## 2. Materials and Methods

### 2.1. Study Population

The Mediterranean healthy Eating, Aging, and Lifestyles (MEAL) study is an observational study designed to explore the relation between nutritional and lifestyle behaviors that characterizes individuals living in the Mediterranean area. The results of a cross-sectional analysis of baseline data are presented in this manuscript. The details of the study protocol, with the rationale, design, and methods, have been described elsewhere [23]. Briefly, the cohort consisted of a random sample of 2044 men and women (age 18+ years) recruited in the urban area of Catania, one of the largest cities of the eastern cost of Sicily, southern Italy, during 2014–2015. All study procedures were carried out in accordance with the Declaration of Helsinki (1989) of the World Medical Association. Participants provided written informed consent, and the study protocol was approved by the ethics committee of the referent health authority.

### 2.2. Data Collection

Data regarding demographic (i.e., age, sex, educational level, and occupational level) and lifestyle characteristics (i.e., physical activity, smoking habits, and drinking habits) were collected. Educational level was categorized as: (i) low (primary/secondary), (ii) medium (high school), and (iii) high (university). Occupational level was classified as: (i) unemployed, (ii) low (unskilled workers), (iii) medium (partially skilled workers), and (iv) high (skilled workers). Physical activity level was assessed using the International Physical Activity Questionnaires (IPAQ) [24], which comprised a set of questionnaires (five domains) on time spent being physically active in the last 7 days that allow us to categorize physical activity as: (i) low, (ii) moderate, and (iii) high. Smoking status was classified as: (i) non-smoker, (ii) ex-smoker, and (iii) current smoker. Alcohol consumption was categorized as (i) none, (ii) moderate drinker (0.1–12 g/day), and (iii) regular drinker (>12 g/day). Anthropometric measurements were performed according to standardized methods [25]. Height of the participant, without shoes with the back square against the wall tape, eyes looking straight ahead, with a right-angle triangle resting on the scalp and against the wall, was measured by a health professional to the nearest 0.5 cm. Body mass index (BMI) was calculated from measured height and weight, and patients were categorized as under/normal weight (BMI <25 kg/m^2^), overweight (BMI 25–29.9 kg/m^2^), and obese (BMI ≥30 kg/m^2^) [26].

### 2.3. Dietary Assessment

Dietary data were collected using a validated food frequency questionnaire (FFQ) consisting of 100 food and drink items representative of the diet during the last 6 months [27,28]. Participants were asked how often, on average, they had consumed foods and drinks included in the FFQ, with nine responses ranging from "never" to "4–5 times per day". Intake of food items characterized by seasonality referred to consumption during the period in which the food was available and then adjusted by its proportional intake over one year. After excluding data on 107 participants with unreliable dietary intakes (<1000 or >6000 kcal/d), data from a total of 1936 individuals were included in the analyses for the present study. Following the identification of the food intake, the energy content as well as the micro-nutrient intake was obtained using standard food composition tables of the Italian Research Center for Foods and Nutrition [29]. The process of the estimation of polyphenol intakes has been previously described in detail [30]. Briefly, data on the polyphenol content in foods were retrieved from the Phenol-Explorer database (www.phenol-explorer.eu) [31], using the most recent version of the database containing data on the effects of cooking and food processing on polyphenol contents in order to apply polyphenol-specific retention factors [32]. Micro-nutrient, macro-nutrient, and polyphenol intake were adjusted for total energy intake (kcal/day) using the residual method [33]. Finally, a Mediterranean diet adherence score was calculated based on a previously published methodology [34] using literature-derived weighted median servings of foods characterizing this dietary pattern (including olive oil, fruit, vegetables, cereals, legumes, fish, and moderate alcohol intake indicating higher adherence and meat and meat-based products, dairy products, and no/excessive alcohol consumption indicating lower adherence; the final score comprised nine food categories with a score ranging from 0 points (lowest adherence) to 18 points (highest adherence) [35].

### 2.4. DII Score

A complete description of the process of developing the DII has been published elsewhere [14,36]. Briefly, the dietary data of the sample were first linked to the world database that provided a robust estimate of a mean and SD for each parameter [14]. This was achieved by subtracting the “standard global mean” from the intake reported via the FFQ and dividing this value by the standard deviation (SD) to obtain “z” scores. To minimize the effect of “right skewing”, these “z” scores were then converted to a centered proportion. The centered proportion for each food parameter for each individual was then multiplied by the respective food parameter effect score (inflammatory potential for each food parameter), which was derived from a literature review, to obtain a food-parameter-specific DII score for an individual. All of the food-parameter–specific DII scores were then summed to create the overall DII score for each participant in the study [14]. Finally, energy-adjusted DII (E-DII) scores were calculated using the density method, wherein all food parameters were converted to per 1000 kcal of nutrients and the same procedure was used to relate individual exposure data to the global energy-adjusted database. The DII was based on a total of 33 food parameters (energy, carbohydrate, protein, total fat, alcohol, fiber, cholesterol, saturated fatty acid, monounsaturated fatty acid, polyunsaturated fatty acid, omega 3, omega 6, vitamin A, vitamin B6, vitamin B12, vitamin C, vitamin D, vitamin E, folic acid, iron, magnesium, zinc, selenium, anthocyanidins, flavan3ols, flavones, flavonols, flavonones, isoflavones, garlic, tea, and onion) that were available for the MEAL cohort. The E-DII score was based on 32 food parameters (all of the previous, except for energy).

### 2.5. Sleep Quality

The Pittsburgh sleep quality index (PSQI) [37] was used to assess participants’ sleep quality and disturbances in the past six months. It consists of 19 items that are rated on a four-point scale (0–3) and grouped into seven components (sleep quality, sleep latency, sleep duration, habitual sleep efficiency, sleep disturbance, use of sleeping medications, and daytime dysfunction). The item scores in each component were summed and converted to component scores ranging from 0 (better) to 3 (worse) based on guidelines. Total PSQI score was calculated as the summation of seven component scores ranging from 0 to 21, where a higher score indicates worse sleep. A score of <5 on total global PSQI score is indicative of adequate sleep quality.

### 2.6. Statistical Analysis

Categorical variables are presented as frequencies of occurrence and percentages, and continuous variables are presented as the mean and standard deviation (SD); differences between groups were tested using a Chi-squared test or a Student’s *t*-test, respectively. The DII was analyzed both as a categorical (quartiles) or a continuous (1 SD increment) variable. The relation between the DII and sleep-related outcomes was tested using a simple univariable (unadjusted) and multivariable logistic regression analysis adjusted for baseline characteristics (age, sex, marital, educational, and occupational status, smoking and alcohol drinking habits, and physical activity level) comparing individuals grouped into quartiles or estimating the association by a 1 SD increment of the DII. Because previous studies suggested a potential role of the Mediterranean diet in sleep quality [19,20] as well as a previous analysis in the cohort [21], we also tested whether the association between DII and sleep quality was independent of adherence to this dietary pattern by performing an additional model that further adjusted for the Mediterranean diet adherence score. All reported *P* values were based on two-sided tests and compared to a significance level of 5%. The SPSS^®^ 17 (SPSS Inc., Chicago, IL, USA) software was used for all of the statistical analyses.

## 3. Results

The distribution of background variables by quartiles of DII is shown in Table 1. There were no clear trends in the distribution of age, sex, weight, or health status across DII quartiles. However, a higher proportion of individuals in lower occupational status categories were found in the lower quartiles of DII, while those with medium–high status had significantly higher DII scores. Moreover, there was a higher proportion of regular alcohol drinkers in the lower quartiles of DII, while none–moderate drinkers had higher DII scores (Table 1).

The frequency of adequate overall sleep quality and its individual domains across quartiles of DII are shown in Table 2. A lower proportion of participants with adequate sleep quality was found among higher quartiles of DII. Among individual domains, a significantly higher rate of sleep disturbance and low self-rated sleep quality was found among participants with higher DII scores (Table 2).

Table 3 provides a direct comparison of mean DII scores between individuals with adequate/inadequate sleep quality features. Overall, the results reflected the previous findings, with average DII scores higher among individuals with inadequate sleep quality, self-rated sleep quality, and, in addition, long sleep latency and efficiency (Table 3).

The association of overall sleep quality and its individual domains with the DII is shown in Table 4. Individuals in the highest quartile of DII were less likely to have adequate sleep quality (odds ratio (*OR*) = 0.49, 95% CI: 0.31, 0.78); the association remained significant also when considering a 1-SD increment of the score (*OR* = 0.73, 95% CI: 0.61, 0.88). Among individual domains of sleep quality, an association with the highest exposure category was found only for sleep latency (*OR* = 0.60, 95% CI: 0.39, 0.93), while the linear association with a 1-SD increment of the score was significant for sleep duration (*OR* = 0.79, 95% CI: 0.66, 0.93; Table 4).

## 4. Discussion

In the present study, the relation between DII and sleep quality was investigated in a cohort of Italian adults. Individuals with higher DII scores were found to be significantly less likely to have adequate sleep quality. Among the various individual components of the sleep quality score, the strongest association was found for sleep latency. Interestingly, after adjusting for adherence to the Mediterranean diet, the association between DII and sleep became even stronger, suggesting that if both the DII and the Mediterranean diet act through a similar mechanism of action related to inflammation, the DII is a stronger predictor for the inflammatory potential of the diet. To our knowledge, this is the first study to focus on the relation between the inflammatory potential of diet and sleep quality parameters.

Existing studies on the role of nutrition in sleep quality by a mediating effect of inflammatory biomarkers are scarce. A study conducted on about 1500 community-dwelling middle-aged men reported that an inverse association between plant-sourced dietary pattern (characterized by beta-carotene, vitamin A, lutein, and zeaxanthin) and CRP was stronger in participants with severe sleep apnoea [38]. In a study exploring the association of sleep with metabolic pathways and metabolites, researchers have found that several metabolites that have previously been linked to inflammation and oxidative stress, including erythrulose (advanced glycation end-product) (positive association) and several γ-glutamyl pathway metabolites, including 3-carboxy-4-methyl-5-propyl-2-furanpropanoic acid (CMPF, fatty acid, dicarboxylate), isovalerate (valine, leucine, and isoleucine and fatty acid metabolism), and inflammation associated complement component 3 peptide (HWESASXX) (inverse association) were associated with sleep parameters (i.e., duration) [39]. Data on about 2000 individuals from the National Health and Nutrition Examination Survey (NHANES) revealed that both healthy eating and adequate sleep were the two health behavior pairs associated with lower levels of inflammation [40]. More in-depth studies conducted on the same cohort showed that adequate sleep quality was associated with optimal inflammation, oxidative stress, and antioxidant level, while selected sleep quality–cardio–metabolic health relationships were moderately mediated by C-reactive protein (CPR) and vitamins A and C; additionally, in women only, the indirect effects were moderate-to-large for CRP, GGT, carotenoids, uric acid, and vitamin C [8]. Specifically, moderate-to-large indirect mediation by GGT, carotenoids, uric acid, and vitamin D was found for sleep duration to waist circumference and systolic blood pressure relationships, whereas vitamin C was a moderate mediator of the sleep duration to diastolic blood pressure relationship [9]. These results suggest that inflammation may be a key mediating effect between sleep-related disorders and other conditions known to be related to a subclinical, low-grade inflammatory status. Chronic alteration of sleep quality has been related to mental health impairment and increased risk of cognitive disorders, including stress, depression, dementia, and Alzheimer’s disease [41]. Diet has been studied over the last few decades for its potential role in affecting mental health. A number of comprehensive reviews of the literature have been performed to investigate the role of diet in affective and cognitive disorders that may be related to sleep impairment. A meta-analysis showed that a high-quality diet, regardless of type (i.e., healthy/prudent or Mediterranean) together with a relatively low dietary inflammatory index was associated with a lower risk of depressive symptoms [42]. Similarly, another meta-analysis investigating a posteriori derived dietary patterns showed that a diet characterized by a high intakes of fruit, vegetables, whole grain, fish, olive oil, low-fat dairy, and antioxidants and low intakes of animal-derived foods was apparently associated with a decreased risk of depression; in contrast, a dietary pattern characterized by a high consumption of red and/or processed meat, refined grains, sweets, high-fat dairy products, butter, potatoes, and high-fat gravy, and low intakes of fruits and vegetables was associated with an increased risk of depression [43]. There also is evidence that a pro-inflammatory diet, as indicated by a higher DII score, may be associated with an increased risk of having depressive symptoms [44]. Regarding cognitive disorders, a systematic review exploring their relation with various dietary patterns showed that the Mediterranean diet had the strongest evidence supporting protection against cognitive decline among older adults. However, studies on the Dietary Approach to Stop Hypertension (DASH) diet, the Mediterranean-DASH diet, the Intervention for Neurodegenerative Delay (MIND) diet, the anti-inflammatory diet and the healthy diet recommended by guidelines via the dietary index, and prudent healthy diets generated via statistical approaches also provided promising results [45]. Moreover, previous studies specifically investigating the inflammatory potential of the diet showed that pro-inflammatory dietary patterns (also identified by higher DII scores) were associated with higher concentrations of inflammatory markers and accelerated cognitive decline at older ages [46,47].

The mechanisms through which diet may influence mental health features include effects on inflammation and oxidative stress, as well as a direct effect through the gut–brain axis. The relation between the DII and mental health may depend, at least in part, on the underlying potential effect of specific foods and compounds on influencing inflammatory pathways. In fact, several dietary factors have been hypothesized to play a role in systemic inflammation [48]. Plant-derived foods are important sources of antioxidants, including vitamins and polyphenols, which have been shown to contribute to inflammatory response and may exert neuroprotective effects and reduce oxidative damage [49]. Healthy fats, such as mono- and certain poly-unsaturated fatty acids, have been shown to play a significant role in neuroinflammation leading to a lower risk of affective disorders and could improve inflammation-associated depressive symptoms [50]. In contrast, food sources of refined carbohydrates may negatively affect dietary glycemic load and index, which have been associated with an acute inflammatory response [51]. Similarly, consumption of meat products has been associated with production of inflammation-provoking antibodies and an increase in pro-inflammatory response [52].

Besides its direct metabolic effects and immunologic responses related to nutritional factors, diet is known to influence the gut microbiota, which may play a role in chronic and low-grade activation of the inflammatory system’s spread from peripheral tissue to the brain [53]. In fact, there is a large body of experimental and clinical studies suggesting a mechanistic link between gut-derived inflammatory response and neurodegeneration, potentially contributing to the pathogenesis of affective and cognitive disorders [54]. This inflammatory status could be triggered by changes in the gut microbiota’s composition and dysbiosis due to dietary habits, i.e., consumption of pro-inflammatory diets, high in fat and sugar, in contrast to high fiber and whole grains, which would lead to an anti-inflammatory response [55,56]. The mechanisms involved in the process have yet to be fully elucidated, but they may include the modulation of plasma levels of lipopolysaccharide, and the inflammasome, type I interferon, and NF-KB (nuclear factor kappa-light-chain-enhancer of activated B cells) signaling pathways [57].

The present study has some limitations that should be kept in mind when considering its results. First, the cross-sectional design does not allow for considering temporality in judging whether a causal relation exists; rather, it provides evidence of an association with no clear cause–effect identification. Thus, reverse-causation should be taken into account; namely, we are not able to determine whether the inflammatory potential of the diet affects sleep quality or sleep features lead to unhealthy dietary habits. Second, the structured assessment methods that were used to assess dietary habits, such as the FFQ, are known to be associated with recall bias [58,59]. However, no ideal method to collect dietary data exists and the FFQ is widely used in nutritional epidemiology.

## 5. Conclusions

In conclusion, these findings confirm our original hypothesis that the inflammatory potential of the diet is associated with sleep quality in adults. Future studies with prospective designs with the potential to provide stronger data for causal inference should be designed and implemented. Interventions to improve diet quality might consider including a dietary component that aims to lower chronic systemic inflammation to prevent cognitive decline and improve sleep quality.

## Figures and Tables

**Table 1:** Baseline characteristics of the study sample according to Dietary Inflammatory Index (DII) quartiles.

	DII Quartiles				*p*-Value
	Q1	Q2	Q3	Q4	
Age groups, *n* (%)					<0.001
<30	95 (19.4)	85 (16.7)	81 (16.1)	89 (20.5)	
30–49	184 (37.6)	169 (33.1)	196 (39.0)	154 (35.4)	
50–69	160 (32.7)	190 (37.3)	166 (33.1)	109 (25.1)	
≥70	50 (10.2)	66 (12.9)	59 (11.8)	83 (19.1)	
Men, *n* (%)	174 (35.4)	235 (46.1)	214 (43.1)	181 (41.4)	0.006
Weight status, *n* (%)					0.01
BMI <25	240 (52.5)	197 (41.9)	202 (44.2)	212 (51.2)	
BMI 25–30	151 (33.0)	179 (38.1)	170 (37.2)	130 (31.4)	
BMI >30	66 (14.4)	94 (20.0)	85 (18.6)	72 (17.4)	
Smoking status, *n* (%)					0.93
Current	120 (24.4)	121 (23.7)	112 (22.5)	112 (25.6)	
Former	67 (13.6)	78 (15.3)	71 (14.3)	60 (13.7)	
Never	305 (62.0)	311 (61.0)	314 (63.2)	265 (60.6)	
Educational level, *n* (%)					0.26
Low	186 (37.8)	189 (37.1)	161 (32.4)	161 (36.8)	
Medium	189 (38.4)	190 (37.3)	192 (38.6)	149 (34.1)	
High	117 (23.8)	131 (25.7)	144 (29.0)	127 (29.1)	
Occupational level, *n* (%)					0.001
Unemployed	145 (34.8)	98 (22.4)	132 (30.8)	86 (22.9)	
Low	70 (16.8)	81 (18.5)	61 (14.3)	54 (14.4)	
Medium	94 (22.5)	127 (29.1)	108 (25.2)	111 (29.5)	
High	108 (25.9)	131 (30.0)	127 (29.7)	125 (33.2)	
Physical activity level, *n* (%)					0.15
Low	92 (21.1)	75 (17.0)	86 (19.1)	76 (18.9)	
Medium	206 (47.2)	209 (47.5)	223 (49.6)	218 (54.2)	
High	138 (31.7)	156 (35.5)	141 (31.3)	108 (26.9)	
Alcohol consumption, *n* (%)					<0.001
None	88 (17.9)	91 (17.8)	107 (21.5)	88 (20.1)	
Moderate (0.1–12 g/day)	252 (51.2)	312 (61.2)	332 (66.8)	310 (70.9)	
Regular (>12 g/day)	152 (30.9)	107 (21.0)	58 (11.7)	39 (8.9)	
Health status, *n* (%)					
Hypertension	220 (44.7)	279 (54.7)	254 (51.1)	223 (51.0)	0.02
Diabetes	34 (6.9)	49 (9.6)	44 (8.9)	19 (4.3)	0.01
Dyslipidemias	101 (20.5)	99 (19.4)	80 (16.1)	76 (17.4)	0.27
Cardiovascular disease	42 (8.7)	34 (7.0)	43 (8.8)	35 (8.1)	0.70
Cancer	24 (4.9)	16 (3.1)	15 (3.0)	23 (5.3)	0.17

**Table 2:** Overall sleep quality and sleep-related characteristics of the study participants by quartiles of Dietary Inflammatory Index (DII).

	DII Quartiles				
	Q1	Q2	Q3	Q4	*p*-Value
Adequate sleep quality, *n* (%)	357 (72.6)	339 (66.5)	340 (68.4)	278 (63.6)	0.03
Sleep duration, *n* (%)					0.71
>7 h	296 (60.2)	316 (62.0)	292 (58.8)	260 (59.5)	
6–7 h	121 (24.6)	108 (21.2)	106 (21.3)	100 (22.9)	
5–6 h	49 (10.0)	58 (11.4)	71 (14.3)	52 (11.9)	
<5 h	26 (5.3)	28 (5.5)	28 (5.6)	25 (5.7)	
Sleep disturbance, *n* (%)					0.04
None	70 (14.2)	53 (10.4)	57 (11.5)	36 (8.2)	
Low	366 (74.4)	373 (73.1)	368 (74.0)	330 (75.5)	
Medium	56 (11.4)	84 (16.5)	72 (14.5)	71 (16.2)	
High	0	0	0	0	
Sleep latency, *n* (%)					0.17
Very short	227 (46.1)	240 (47.1)	221 (44.5)	170 (38.9)	
Short	157 (31.9)	152 (29.8)	173 (34.8)	147 (33.6)	
Medium	83 (16.9)	85 (16.7)	79 (15.9)	86 (19.7)	
Long	25 (5.1)	33 (6.5)	24 (4.8)	34 (7.8)	
Day dysfunction due to sleepiness, *n* (%)					0.30
None	300 (61.0)	273 (53.5)	273 (54.9)	234 (53.5)	
Low	163 (33.1)	196 (38.4)	181 (36.4)	159 (36.4)	
Medium	27 (5.5)	39 (7.6)	41 (8.2)	42 (9.6)	
High	2 (0.4)	2 (0.4)	2 (0.4)	2 (0.5)	
Sleep efficiency, *n* (%)					0.27
High	360 (73.2)	365 (71.6)	342 (68.8)	303 (69.3)	
Medium	64 (13.0)	75 (14.7)	82 (16.5)	68 (15.6)	
Low	27 (5.5)	32 (6.3)	21 (4.2)	33 (7.6)	
Very low	41 (8.3)	38 (7.5)	52 (10.5)	33 (7.6)	
Self-rated sleep quality, *n* (%)					<0.05
Very low	16 (3.3)	16 (3.1)	13 (2.6)	21 (4.8)	
Low	57 (11.6)	91 (17.8)	70 (14.1)	61 (14.0)	
Medium	301 (61.2)	286 (56.1)	307 (61.8)	276 (63.2)	
High	118 (24.0)	117 (22.9)	107 (21.5)	79 (18.1)	
Need of medication to sleep, *n* (%)					0.44
Not during the past month	453 (92.1)	453 (88.8)	447 (89.9)	391 (89.5)	
Less than once a week	16 (3.3)	23 (4.5)	20 (4.0)	15 (3.4)	
Once or twice a week	5 (1.0)	13 (2.5)	16 (3.2)	13 (3.0)	
Three or more times a week	18 (3.7)	21 (4.1)	14 (2.8)	18 (4.1)	

**Table 3:** Mean scores (and standard deviation) of Dietary Inflammatory Index (DII) by outcome.

	DII, Mean (SD)	*p*-Value
Sleep quality		<0.001
Inadequate	−0.67 (2.09)	
Adequate	−1.05 (2.10)	
Sleep duration		0.16
Inadequate	−0.85 (2.09)	
Adequate	−0.98 (2.10)	
Sleep disturbance		0.006
Inadequate	−0.88 (2.11)	
Adequate	−1.30 (1.96)	
Sleep latency		0.01
Inadequate	−0.82 (2.10)	
Adequate	−1.06 (2.10)	
Day dysfunction		0.09
Inadequate	−0.84 (2.08)	
Adequate	−1.00 (2.11)	
Sleep efficiency		0.01
Inadequate	−0.74 (2.06)	
Adequate	−1.00 (2.11)	
Need of medication to sleep		0.14
Inadequate	−0.72 (1.95)	
Adequate	−0.95 (2.11)	
Self-rated sleep quality		0.006
Inadequate	−0.86 (2.12)	
Adequate	−1.18 (2.00)	

**Table 4:** Association between overall and individual domains of sleep quality ^†^ by quartiles of the DII.

	DII, *OR* (95% CI)				1-SD Increment
	Q1	Q2	Q3	Q4	
Adequate sleep quality					
Model 1 ^‡^	1	0.67 (0.51, 0.88)	0.65 (0.49, 0.86)	0.57 (0.43, 0.75)	0.83 (0.75, 0.92)
Model 2 ^§^	1	0.76 (0.54, 1.07)	0.79 (0.56, 1.11)	0.67 (0.47, 0.95)	0.86 (0.76, 0.97)
Model 3 ^¶^	1	0.67 (0.47, 0.96)	0.65 (0.44, 0.96)	0.49 (0.31, 0.78)	0.73 (0.61, 0.88)
Sleep duration					
Model 1 ^‡^	1	1.02 (0.79, 1.31)	0.84 (0.65, 1.08)	0.89 (0.68, 1.15)	0.93 (0.85, 1.03)
Model 2 ^§^	1	1.19 (0.86, 1.64)	1.00 (0.73, 1.37)	1.00 (0.72, 1.38)	0.95 (0.85, 1.07)
Model 3 ^¶^	1	1.04 (0.75, 1.46)	0.77 (0.53, 1.12)	0.67 (0.43, 1.04)	0.79 (0.66, 0.93)
Sleep disturbance					
Model 1 ^‡^	1	0.66 (0.45, 0.96)	0.73 (0.50, 1.06)	0.52 (0.34, 0.80)	0.81 (0.69, 0.94)
Model 2 ^§^	1	0.73 (0.46, 1.16)	0.76 (0.48, 1.20)	0.56 (0.34, 0.93)	0.82 (0.69, 0.98)
Model 3 ^¶^	1	0.73 (0.45, 1.19)	0.79 (0.46, 1.35)	0.62 (0.31, 1.21)	0.84 (0.65, 1.09)
Sleep latency					
Model 1 ^‡^	1	0.95 (0.74, 1.22)	0.83 (0.65, 1.07)	0.69 (0.53, 0.90)	0.89 (0.81, 0.97)
Model 2 ^§^	1	1.00 (0.73, 1.36)	0.87 (0.64, 1.19)	0.73 (0.53, 1.01)	0.90 (0.80, 1.01)
Model 3 ^¶^	1	0.93 (0.67, 1.28)	0.77 (0.54, 1.10)	0.60 (0.39, 0.93)	0.85 (0.72, 1.00)
Day dysfunction					
Model 1 ^‡^	1	0.70 (0.55, 0.90)	0.73 (0.57, 0.94)	0.70 (0.54, 0.91)	0.92 (0.84, 1.01)
Model 2 ^§^	1	0.67 (0.49, 0.92)	0.75 (0.54, 1.02)	0.66 (0.48, 0.91)	0.90 (0.80, 1.01)
Model 3 ^¶^	1	0.67 (0.48, 0.94)	0.76 (0.53, 1.10)	0.68 (0.44, 1.06)	0.94 (0.79, 1.11)
Sleep efficiency					
Model 1 ^‡^	1	0.85 (0.64, 1.12)	0.68 (0.52, 0.90)	0.74 (0.55, 0.99)	0.88 (0.79, 0.97)
Model 2 ^§^	1	0.91 (0.64, 1.29)	0.79 (0.56, 1.12)	0.87 (0.61, 1.23)	0.91 (0.80, 1.04)
Model 3 ^¶^	1	0.88 (0.61, 1.27)	0.75 (0.50, 1.11)	0.78 (0.48, 1.25)	0.85 (0.71, 1.02)
Need of medication to sleep					
Model 1 ^‡^	1	0.63 (0.41, 0.98)	0.67 (0.43, 1.05)	0.67 (0.43, 1.06)	0.89 (0.76, 1.04)
Model 2 ^§^	1	0.72 (0.42, 1.24)	0.84 (0.48, 1.45)	0.92 (0.52, 1.63)	0.99 (0.81, 1.21)
Model 3 ^¶^	1	0.67 (0.38, 1.17)	0.76 (0.40, 1.44)	0.79 (0.37, 1.69)	0.92 (0.69, 1.22)
Self-rated sleep quality					
Model 1 ^‡^	1	0.92 (0.69, 1.22)	0.78 (0.58, 1.05)	0.65 (0.47, 0.89)	0.85 (0.76, 0.96)
Model 2 ^§^	1	1.10 (0.76, 1.58)	0.99 (0.69, 1.44)	0.75 (0.50, 1.11)	0.90 (0.78, 1.03)
Model 3 ^¶^	1	1.18 (0.81, 1.72)	1.15 (0.75, 1.77)	0.97 (0.58, 1.64)	1.01 (0.83, 1.23)

^†^ Higher scores indicate worse quality. ^‡^ Model 1 is unadjusted for any covariate. ^§^ Model 2 is adjusted for age (continuous), sex (male/female), body mass index (BMI, <25 kg/m2, 25–30 kg/m2, >30 kg/m2), physical activity (low/medium/high), educational status (low/medium/high), occupational status (unemployed/low/medium/high), smoking status (current/former/never), alcohol consumption (no/moderate/regular), health status (presence of hypertension, type-2 diabetes, dyslipidemias, cardiovascular disease, cancer), and total energy intake. ^¶^ Model 3 adjusted as in Model 2 + adherence to Mediterranean diet.

## References

[1] Pawelec G., Goldeck D., Derhovanessian E. (2014). Inflammation, ageing and chronic disease. Curr. Opin. Immunol..

[2] Prasad S., Sung B., Aggarwal B.B. (2012). Age-associated chronic diseases require age-old medicine: Role of chronic inflammation. Prev. Med..

[3] Cunningham C. (2013). Microglia and neurodegeneration: The role of systemic inflammation. Glia.

[4] Nuzzo D., Picone P., Caruana L., Vasto S., Barera A., Caruso C., Di Carlo M. (2014). Inflammatory mediators as biomarkers in brain disorders. Inflammation.

[5] Clark I.A., Vissel B. (2014). Inflammation-sleep interface in brain disease: Tnf, insulin, orexin. J. Neuroinflamm..

[6] Everson C.A., Laatsch C.D., Hogg N. (2005). Antioxidant defense responses to sleep loss and sleep recovery. Am. J. Physiol. Regul. Integr. Comp. Physiol..

[7] Ng A., Tam W.W., Zhang M.W., Ho C.S., Husain S.F., McIntyre R.S., Ho R.C. (2018). Il-1beta, il-6, tnf-alpha and crp in elderly patients with depression or alzheimer’s disease: Systematic review and meta-analysis. Sci. Rep..

[8] Kanagasabai T., Ardern C.I. (2015). Inflammation, oxidative stress, and antioxidants contribute to selected sleep quality and cardiometabolic health relationships: A cross-sectional study. Mediat. Inflamm..

[9] Kanagasabai T., Ardern C.I. (2015). Contribution of inflammation, oxidative stress, and antioxidants to the relationship between sleep duration and cardiometabolic health. Sleep.

[10] Soory M. (2012). Nutritional antioxidants and their applications in cardiometabolic diseases. Infect. Disord. Drug Targets.

[11] Barbaresko J., Koch M., Schulze M.B., Nothlings U. (2013). Dietary pattern analysis and biomarkers of low-grade inflammation: A systematic literature review. Nutr. Rev..

[12] Neale E.P., Batterham M.J., Tapsell L.C. (2016). Consumption of a healthy dietary pattern results in significant reductions in c-reactive protein levels in adults: A meta-analysis. Nutr. Res..

[13] Schwingshackl L., Hoffmann G. (2014). Mediterranean dietary pattern, inflammation and endothelial function: A systematic review and meta-analysis of intervention trials. Nutr. Metab. Cardiovasc. Dis..

[14] Shivappa N., Steck S.E., Hurley T.G., Hussey J.R., Hebert J.R. (2014). Designing and developing a literature-derived, population-based dietary inflammatory index. Public Health Nutr..

[15] Fowler M.E., Akinyemiju T.F. (2017). Meta-analysis of the association between dietary inflammatory index (dii) and cancer outcomes. Int. J. Cancer.

[16] Namazi N., Larijani B., Azadbakht L. (2018). Dietary inflammatory index and its association with the risk of cardiovascular diseases, metabolic syndrome, and mortality: A systematic review and meta-analysis. Horm. Metab. Res..

[17] Shivappa N., Godos J., Hebert J.R., Wirth M.D., Piuri G., Speciani A.F., Grosso G. (2018). Dietary inflammatory index and cardiovascular risk and mortality—A meta-analysis. Nutrients.

[18] Shivappa N., Bonaccio M., Hebert J.R., Di Castelnuovo A., Costanzo S., Ruggiero E., Pounis G., Donati M.B., de Gaetano G., Iacoviello L. (2018). Association of proinflammatory diet with low-grade inflammation: Results from the moli-sani study. Nutrition.

[19] Campanini M.Z., Guallar-Castillon P., Rodriguez-Artalejo F., Lopez-Garcia E. (2017). Mediterranean diet and changes in sleep duration and indicators of sleep quality in older adults. Sleep.

[20] Jaussent I., Dauvilliers Y., Ancelin M.L., Dartigues J.F., Tavernier B., Touchon J., Ritchie K., Besset A. (2011). Insomnia symptoms in older adults: Associated factors and gender differences. Am. J. Geriatr. Psychiatry.

[21] Godos J., Ferri R., Caraci F., Cosentino F.I.I., Castellano S., Galvano F., Grosso G. (2019). Adherence to the mediterranean diet is associated with better sleep quality in italian adults. Nutrients.

[22] Shin D., Kwon S.C., Kim M.H., Lee K.W., Choi S.Y., Shivappa N., Hebert J.R., Chung H.K. (2018). Inflammatory potential of diet is associated with cognitive function in an older adult korean population. Nutrition.

[23] Grosso G., Marventano S., D‘Urso M., Mistretta A., Galvano F. (2017). The mediterranean healthy eating, ageing, and lifestyle (meal) study: Rationale and study design. Int. J. Food Sci. Nutr..

[24] Craig C.L., Marshall A.L., Sjostrom M., Bauman A.E., Booth M.L., Ainsworth B.E., Pratt M., Ekelund U., Yngve A., Sallis J.F. (2003). International physical activity questionnaire: 12-country reliability and validity. Med. Sci. Sports Exerc..

[25] Mistretta A., Marventano S., Platania A., Godos J., Galvano F., Grosso G. (2017). Metabolic profile of the mediterranean healthy eating, lifestyle and aging (meal) study cohort. Mediterr. J. Nutr. Metab..

[26] World Health Organization (1997). Obesity: Preventing and Managing the Global Epidemic: Report of a Who Consultation on Obesity, Geneva, The Switzerland, 3–5 June 1997.

[27] Marventano S., Mistretta A., Platania A., Galvano F., Grosso G. (2016). Reliability and relative validity of a food frequency questionnaire for italian adults living in sicily, southern italy. Int. J. Food Sci. Nutr..

[28] Buscemi S., Rosafio G., Vasto S., Massenti F.M., Grosso G., Galvano F., Rini N., Barile A.M., Maniaci V., Cosentino L. (2015). Validation of a food frequency questionnaire for use in italian adults living in sicily. Int. J. Food Sci. Nutr..

[29] Istituto Nazionale di Ricerca per gli Alimenti e la Nutrizione (2009). Tabelle di Composizione Degli Alimenti.

[30] Godos J., Marventano S., Mistretta A., Galvano F., Grosso G. (2017). Dietary sources of polyphenols in the mediterranean healthy eating, aging and lifestyle (meal) study cohort. Int. J. Food Sci. Nutr..

[31] Neveu V., Perez-Jiménez J., Vos F., Crespy V., du Chaffaut L., du Chaffaut L., Mennen L., Knox C., Eisner R., Cruz J. (2010). Phenol-explorer: An online comprehensive database on polyphenol contents in foods. Database.

[32] Rothwell J.A., Perez-Jimenez J., Neveu V., Medina-Remon A., M’Hiri N., Garcia-Lobato P., Manach C., Knox C., Eisner R., Wishart D.S. (2013). Phenol-explorer 3.0: A major update of the phenol-explorer database to incorporate data on the effects of food processing on polyphenol content. Database.

[33] Willett W.C.L.E. (1998). Reproducibility and validity of food frequency questionnaire. Nutritional Epidemiology.

[34] Marventano S., Godos J., Platania A., Galvano F., Mistretta A., Grosso G. (2018). Mediterranean diet adherence in the mediterranean healthy eating, aging and lifestyle (meal) study cohort. Int. J. Food Sci. Nutr..

[35] Sofi F., Macchi C., Abbate R., Gensini G.F., Casini A. (2014). Mediterranean diet and health status: An updated meta-analysis and a proposal for a literature-based adherence score. Public Health Nutr..

[36] Cavicchia P.P., Steck S.E., Hurley T.G., Hussey J.R., Ma Y., Ockene I.S., Hebert J.R. (2009). A new dietary inflammatory index predicts interval changes in serum high-sensitivity c-reactive protein. J. Nutr..

[37] Buysse D.J., Reynolds C.F., Monk T.H., Berman S.R., Kupfer D.J. (1989). The pittsburgh sleep quality index: A new instrument for psychiatric practice and research. Psychiatry Res..

[38] Cao Y., Wittert G., Taylor A.W., Adams R., Appleton S., Shi Z. (2017). Nutrient patterns and chronic inflammation in a cohort of community dwelling middle-aged men. Clin. Nutr..

[39] Gordon-Dseagu V.L.Z., Derkach A., Xiao Q., Williams I., Sampson J., Stolzenberg-Solomon R.Z. (2019). The association of sleep with metabolic pathways and metabolites: Evidence from the dietary approaches to stop hypertension (dash)-sodium feeding study. Metabolomics.

[40] Loprinzi P.D. (2016). Health behavior combinations and their association with inflammation. Am. J. Health Promot..

[41] Pace-Schott E.F., Spencer R.M. (2015). Sleep-dependent memory consolidation in healthy aging and mild cognitive impairment. Curr. Top. Behav. Neurosci..

[42] Molendijk M., Molero P., Ortuno Sanchez-Pedreno F., Van der Does W., Angel Martinez-Gonzalez M. (2018). Diet quality and depression risk: A systematic review and dose-response meta-analysis of prospective studies. J. Affect. Disord..

[43] Li Y., Lv M.R., Wei Y.J., Sun L., Zhang J.X., Zhang H.G., Li B. (2017). Dietary patterns and depression risk: A meta-analysis. Psychiatry Res..

[44] Wang J., Zhou Y., Chen K., Jing Y., He J., Sun H., Hu X. (2018). Dietary inflammatory index and depression: A meta-analysis. Public Health Nutr..

[45] Chen X., Maguire B., Brodaty H., O’Leary F. (2019). Dietary patterns and cognitive health in older adults: A systematic review. J. Alzheimers Dis..

[46] Hayden K.M., Beavers D.P., Steck S.E., Hebert J.R., Tabung F.K., Shivappa N., Casanova R., Manson J.E., Padula C.B., Salmoirago-Blotcher E. (2017). The association between an inflammatory diet and global cognitive function and incident dementia in older women: The women‘s health initiative memory study. Alzheimers Dement..

[47] Ozawa M., Shipley M., Kivimaki M., Singh-Manoux A., Brunner E.J. (2017). Dietary pattern, inflammation and cognitive decline: The whitehall ii prospective cohort study. Clin. Nutr..

[48] Minihane A.M., Vinoy S., Russell W.R., Baka A., Roche H.M., Tuohy K.M., Teeling J.L., Blaak E.E., Fenech M., Vauzour D. (2015). Low-grade inflammation, diet composition and health: Current research evidence and its translation. Br. J. Nutr..

[49] Ayuso M.I., Gonzalo-Gobernado R., Montaner J. (2017). Neuroprotective diets for stroke. Neurochem. Int..

[50] Marventano S., Kolacz P., Castellano S., Galvano F., Buscemi S., Mistretta A., Grosso G. (2015). A review of recent evidence in human studies of n-3 and n-6 pufa intake on cardiovascular disease, cancer, and depressive disorders: Does the ratio really matter?. Int. J. Food Sci. Nutr..

[51] Beilharz J.E., Maniam J., Morris M.J. (2015). Diet-induced cognitive deficits: The role of fat and sugar, potential mechanisms and nutritional interventions. Nutrients.

[52] Alisson-Silva F., Kawanishi K., Varki A. (2016). Human risk of diseases associated with red meat intake: Analysis of current theories and proposed role for metabolic incorporation of a non-human sialic acid. Mol. Aspects Med..

[53] Albenberg L.G., Wu G.D. (2014). Diet and the intestinal microbiome: Associations, functions, and implications for health and disease. Gastroenterology.

[54] Kowalski K., Mulak A. (2019). Brain-gut-microbiota axis in alzheimer’s disease. J. Neurogastroenter. Motil..

[55] Solas M., Milagro F.I., Ramirez M.J., Martinez J.A. (2017). Inflammation and gut-brain axis link obesity to cognitive dysfunction: Plausible pharmacological interventions. Curr. Opin. Pharmacol..

[56] Telle-Hansen V.H., Holven K.B., Ulven S.M. (2018). Impact of a healthy dietary pattern on gut microbiota and systemic inflammation in humans. Nutrients.

[57] Ma Q., Xing C., Long W., Wang H.Y., Liu Q., Wang R.F. (2019). Impact of microbiota on central nervous system and neurological diseases: The gut-brain axis. J. Neuroinflamm..

[58] Hebert J.R., Clemow L., Pbert L., Ockene I.S., Ockene J.K. (1995). Social desirability bias in dietary self-report may compromise the validity of dietary intake measures. Int. J. Epidemiol..

[59] Hebert J.R., Ma Y., Clemow L., Ockene I.S., Saperia G., Stanek E.J., Merriam P.A., Ockene J.K. (1997). Gender differences in social desirability and social approval bias in dietary self-report. Am. J. Epidemiol..

